# An Assessment of the Oral and Inhalation Acute Toxicity of Nickel Oxide Nanoparticles in Rats

**DOI:** 10.3390/nano13020261

**Published:** 2023-01-07

**Authors:** Tara Lyons-Darden, Jason L. Blum, Mark W. Schooley, Melissa Ellis, Jennifer Durando, Daniel Merrill, Adriana R. Oller

**Affiliations:** 1 NiPERA Inc., 2525 Meridian Pkwy Ste 240, Durham, NC 27713, USA; 2 Product Safety Labs, 2394 US Highway 130, Dayton, NJ 08810, USA; 3 Oller Consulting, 722 Gaston Manor Dr, Durham, NC 27703, USA

**Keywords:** nickel, nickel oxide, nanoparticle, acute toxicity, nanotoxicity

## Abstract

Nickel oxide nanoparticles (NiO NPs) have been the focus of many toxicity studies. However, acute toxicity studies that identify toxicological dose descriptors, such as an LC_50_ or LD_50_, are lacking. In this paper, the acute toxicity of NiO NPs was evaluated in albino-derived Sprague-Dawley rats through OECD guideline studies conducted by both the oral and inhalation routes of exposure. The animals were assessed for mortality, body weight, behavioral observations, and gross necropsy. Results from previously conducted (unpublished) acute inhalation studies with larger NiO microparticles (MPs) are also included for comparison. Mortality, the primary endpoint in acute toxicity studies, was not observed for rats exposed to NiO NPs via either the oral or inhalation exposure routes, with a determined LD_50_ of >5000 mg/kg and an LC_50_ > 5.42 mg/L, respectively. Our results suggest that these NiO NPs do not exhibit serious acute toxicity in rats or warrant an acute toxicity classification under the current GHS classification criteria. This aligns with similar results for NiO MPs from this and previously published studies.

## 1. Introduction

Nickel (Ni) is an abundant natural element that is essential for plants and thus is naturally found in many of the foods that we safely consume [1,2,3,4]. Additionally, nickel’s physical and chemical properties contribute to the widespread utilization of Ni, Ni compounds, and Ni alloys in many industrial and commercial applications. With this level of exposure, it is no surprise that general Ni toxicity has been widely studied, with the release of Ni^2+^ ions and their ability to interact with specific biological targets (cells, DNA, etc.) identified as the primary source of toxicity. Many studies have shown nickel’s primary association with allergic contact dermatitis after skin contact as well as respiratory effects such as toxicity and/or carcinogenicity after inhalation exposure [5,6]. While established toxicological profiles are available for the traditional Ni-containing particles of various water insoluble Ni substances, this is not the case for much smaller Ni-containing nanomaterials (NMs).

Nanomaterials are defined as having at least one dimension between 1 and 100 nm [7,8]. Their unique size-dependent properties have contributed to a considerable increase in the production and use of engineered nanoparticles (NPs), including Ni NPs, for various applications (e.g., electronics, textiles, aviation, catalysis, etc.) in recent decades [8,9,10,11]. At the nanoscale, many of the physicochemical properties differ from larger bulk-sized or microscale for the same materials [7,8]. Greater surface area, higher surface energy, and magnetism are just some of the properties that make NMs advantageous in a wide variety of products such as electronic circuits, surface coatings, and other technologies [8,12,13,14]. Within these specialized consumer products, NMs are usually tightly embedded, with minimal exposure risk expected for the general public [12,15,16]. However, the increased utilization of these NMs has raised some concern about their potential effects on health and safety with regard to occupational exposures arising from the handling, processing, and analyzing of NMs [13,15,17].

The health effects data are overwhelmingly lacking for many NMs, specifically OECD guideline studies used to establish toxicological dose descriptors. This is true for nickel-based NPs, including nickel oxide (NiO) NPs. Many in vitro and in vivo studies evaluating a multitude of health effects have been conducted with NiO NPs. Acute and chronic studies have shown that NiO NPs are associated with increased lung injury, inflammation, oxidative stress, apoptosis, and altered biochemical indices [18,19,20,21,22,23,24,25]. A detailed analysis of these particular endpoints with considerations of various exposure routes, exposure durations, animal species, etc., is beyond the scope of this paper. However, several recently published literature reviews have evaluated many of these studies and the potential health effects of oxidic Ni NPs [26,27,28]. The acute in vivo studies evaluated in these reviews primarily focused on inflammation, histopathology, cytotoxicity, genotoxicity, oxidative stress, apoptosis, hematopoietic effects, and functional/biochemical indices. Yet, most of these reports do not provide enough information to determine toxicological dose descriptors for key acute toxicity endpoints that can be used for the eventual hazard classification of NPs.

This paper presents data assessing the acute toxicity of engineered NiO NPs and identifies dose descriptors for acute toxicity via both the oral and inhalation routes of exposure. The exposure concentrations selected for these in vivo animal studies are admittedly much higher than any expected acute human exposure [29,30,31]. However, the selected exposure concentrations are aligned with the current criteria for the Globally Harmonized System of Classification and Labelling of Chemicals (GHS), which would likely be used to assign a hazard classification to these NPs. Additionally, the results from the acute oral and inhalation animal studies with NiO NPs are compared to previously conducted studies that evaluated larger non-nanoscale (micron) NiO microparticles (MPs), which are more widely used and have a more established toxicological profile. Specifically, NiO MP data from a previous acute oral study [32] as well as data from acute inhalation studies (performed in 2010 and 2013 but not previously published) are presented in this paper for comparison with the NiO NP results. The study designs and protocols implemented for the NPs are very similar to the previous studies conducted with the MPs.

## 2. Materials and Methods

### 2.1. Test Substances

A commercially available 20 nm engineered NiO NP was chosen and purchased from Nanoshel, LLC (County Cavan, Ireland; Product # NS6130-03-337). A non-nanoscale (micron) NiO black powder (provided by a member of the Nickel REACH Consortia) was also evaluated. The NiO NPs and MPs were stored at room temperature in amber glass bottles topped with nitrogen. Table 1 lists the physicochemical properties of these NiO test substances.

### 2.2. Animal Care and Observations

All animal studies were conducted at Product Safety Labs (Dayton, New Jersey, USA) under animal use protocols approved by Product Safety Labs’ Institutional Animal Care and Use Committee. Albino-derived Sprague-Dawley rats between 8 and 12 weeks of age were obtained from several different sources (see Table 2), as these studies were conducted in different time periods. The rats were housed according to the *Guide for the Care and Use of Laboratory Animals* [33,34], with a 12 h light/dark photoperiod cycle and acclimated to their new environment for 7–9 days or 26–39 days for the MP and NP studies, respectively. Laboratory conditions were within the OECD guideline recommendations during exposures, with reported temperatures between 20 and 25 °C, and 50–70% relative humidity. During the 14-day observation period following exposures, in most cases the temperature and humidity remained within OECD guideline recommendations. However, the humidity was elevated up to 86% for 6 days of the 14-day observation period following NP inhalation exposure. A portable dehumidifier was utilized to lower the high humidity levels resulting from facility deviations during the summer months. The elevated humidity conditions were considered minor, with no impact on the NP study. The rats were provided ad libitum access to food and filtered tap water, except no access to food was available when the rats were fasted overnight prior to oral exposure or during inhalation exposure.

During the 14-day observation period, animals were examined daily for mortality, signs of gross toxicity, and behavioral changes. Individual animal body weight was recorded prior to administration of NiO NPs or MPs on observation days 7 and 14 for the oral study and on observation days 1, 3, 7, and 14 for the inhalation studies. At the end of the 14-day observation period, the rats were euthanized via CO_2_ inhalation and their internal organs were visually examined for gross abnormalities.

### 2.3. Acute Oral Study

The acute oral toxicity study was conducted with six female rats (nulliparous and non-pregnant) according to the OECD 425 Up-And-Down Procedure. Single treatment dose progressions via oral gavage were conducted one at a time with careful evaluation of the animal(s) before moving on to a lower or higher dose. An initial administration of 175 mg/kg (administered as 50% *w*/*w* in distilled water) was given to a female rat, with the upper limit for higher doses capped at 5000 mg/kg. LD_50_s above this dose do not result in acute oral toxicity classification according to GHS hazard classification criteria. All animals were observed for 14 days following oral administration of NiO NPs. A median lethal oral dose (LD_50_), indicating 50% mortality, was determined.

### 2.4. Acute Inhalation Studies

The nose-only exposure studies were conducted using male and female (nulliparous and non-pregnant) rats according to the OECD 403 Acute Inhalation Toxicity guidelines. A total of 40 rats were exposed to the NiO NPs and 20 rats were exposed to the NiO MPs. The NiO NPs and high dose MP powders (8 mg/L) were aerosolized with a Fluid Energy Jet Mill (Schenck Process, Whitewater, WI, USA; Serial J2724E), while a modified Wright Dust Generator (Product Safety Labs, Dayton, NJ, USA; Model # 4Z538A) was used to generate the lower dose (5 mg/L) of MPs. Chamber concentration measurements and particle size distributions were obtained from weighing glass fiber filters (and filter paper in the case of particle size distribution for the MPs) before and after collection of breathing zone samples from the animals at various intervals per exposure. The inhalation study with NiO NPs included exposure to aerosolized NiO NPs for 4 h at a 5 mg/L target concentration. LC_50_s above this concentration do not result in acute inhalation toxicity classification according to GHS hazard classification criteria. The study was carried out in two separate rounds that included a total of 40 animals. The inhalation studies with NiO MPs evaluated single exposures for 4 h at target concentrations of 5 and 8 mg/L. The studies were carried out in two rounds conducted 3 years apart (initial round with 5 mg/L and second round with 8 mg/L). All animals were observed for 14 days following inhalation. A median lethal inhalation concentration (LC_50_), indicating 50% mortality, was determined.

## 3. Results

### 3.1. Acute Oral Toxicity

Following a single oral exposure to NiO NPs at increasing doses from 175 to 5000 mg/kg, no mortality was observed following the 14-day observation period, resulting in an LD_50_ of >5000 mg/kg (Table 3). Additionally, no significant differences in body weights were reported. Individual body weight observations following acute oral exposure to NiO NPs are presented in Supplemental Information, Table S1. All animals appeared to be active and healthy during in-life behavioral observations, with no gross abnormalities observed during necropsy (Supplemental Information, Table S3).

Previously published data with NiO MPs [32] can be compared to the NP data. Following oral exposure to NiO MPs at increasing doses from 5000 to 11,000 mg/kg, mortality was observed during the 14-day observation period at doses of 9990 mg/kg and higher, resulting in an LD_50_ of 9990 mg/kg (Table 3). Individual body weight observations following acute oral exposure to NiO MPs are presented in Supplemental Information, Table S2. At doses below 9990 mg/kg, all animals appeared to be active and healthy during in-life behavioral observations with no gross abnormalities observed during necropsy. However, at doses of 9990 mg/kg and above, several behavioral and gross abnormalities were observed including: reduced fecal volume, hypoactivity, facial staining, and discolored intestines (Supplemental Information, Table S4).

### 3.2. Acute Inhalation Toxicity

Following a single inhalation exposure (4 hrs) to NiO NPs at 5 mg/L in two subsequent rounds, no mortality was observed during the 14-day observation period, with the aggregated data resulting in an LC_50_ of >5.42 mg/L (Table 4). Similarly, single inhalation exposures (4 hrs) to NiO MPs at target levels of 5 and 8 mg/L did not result in mortality, leading to an LC_50_ of >8.30 mg/L. Despite the small nanoscale size of the primary NPs, the mass median aerodynamic diameter (MMAD) of the aerosolized NPs ranged from 3 to 4 μm, similar in size to the MMAD of the MPs. Reduced individual body weights were observed during the first 7 days following inhalation for most animals, with resumed weight gain by the end of the observation period (Supplemental Information, Tables S5 and S6 for NPs and MPs, respectively). For exposures to NiO NPs and MPs, the most common in-life behavioral observations were irregular respiration and hypoactivity during the 14-day observation period and slight to moderate lung discoloration was often observed during necropsy (Supplemental Information, Tables S7 and S8 for NPs and MPs, respectively).

## 4. Discussion

Acute oral and inhalation studies with NiO NPs were conducted to determine their classification for acute toxicity within the current regulatory framework. These studies evaluated doses that align with those needed for GHS hazard classifications for oral and inhalation toxicity. Both the oral and inhalation studies with NPs resulted in no mortality at any of the exposure levels tested. These results are similar to the previously reported oral [32] and currently reported inhalation results for non-nanoscale NiO MPs. Exposure to NiO MPs also did not result in mortality at similar concentrations, though NiO MPs did induce mortality at much higher oral concentrations. Our results indicate that NiO NPs (as with their micron size counterparts) would not warrant an acute toxicity classification under the current GHS classification criteria, an indication of no acute toxicity or hazard concern for single oral or 4 h inhalation exposures.

The results of our oral and inhalation studies, tested at much higher exposure concentrations than expected for human exposure [29,30,31], did not indicate acute toxicity in the form of mortality or other serious effects for NPs, although irregular respiration and discolored lungs were noted in the inhalation study. These results align with our observations for MPs at similar doses. However, at even higher doses than evaluated for the NPs, additional adverse effects were observed for the MPs, including mortality. An important point to note is that the behavioral observations and gross abnormalities noted for the NPs did not differ from those observed for the MPs, indicating no new nano-specific effects of concern. This aligns with other research that suggested similar toxicity profiles for NPs and their non-nanoscale counterparts [26,35]. Additionally, many other studies reporting adverse effects (e.g., inflammation, cytotoxicity, reactive oxygen species or ROS, altered biochemical indices, etc.) for nickel NPs did not always put the results into perspective to indicate if or how they differed from their non-nanoscale counterparts. Further, many in vivo studies that reported adverse effects for nickel NPs used exposure routes that are not relevant for potential human exposure (e.g., intratracheal instillation, oropharyngeal aspiration, and intraperitoneal injection). Our results show that relevant exposure routes with very high exposure concentrations (based on GHS hazard criteria) of NiO NPs do not result in severe acute toxicity or new nano-specific effects. Similar results are to be expected for the much lower exposure concentrations relevant for potential human exposure.

One possible explanation for the similarity of the in vivo results observed for the MPs and NPs may be the tendency of NPs to agglomerate or aggregate to sizes similar to those of MPs [17,36]. Nanoparticle agglomeration is also supported by the measured zeta potential (−14.6 mV) of our NiO NPs, which is within the range to suggest colloidal instability and the increased likelihood of agglomeration or aggregation [37]. Additionally, typical nickel toxicity for non-nanoscale MPs is related to the release of nickel ions and the general low solubility of NiO renders it less likely to release nickel ions compared to more soluble nickel compounds [38,39]. This could be the same for NiO NPs and explain the comparable in vivo results with NiO MPs, even though there is often speculation that NPs have the potential to be more toxic than their non-nanoscale counterparts due to unique size-specific characteristics. There is a general assumption of higher surface reactivity (as measured by ROS) and larger surface area for NPs [40,41,42,43], yet the latter did not hold true for the NiO NPs evaluated in this study. The characterization of the NiO particles (as received) based on a nitrogen adsorption technique indicated that the MPs and NPs had similar surface areas; however, this information did not match the quite different median hydrodynamic diameters measured in water (NPs) and isopar G (MPs). It is uncertain if the surface areas remain similar in vivo once ingested or inhaled, but the similar MMADs indicate that this may be the case at least for inhalation. Interestingly, the high surface reactivity of our NiO NPs (23.8 nM), as measured by ROS, would seem to suggest that the nanoparticles are quite reactive with possibly increased dissolution that would in turn result in a greater likelihood for toxicity. However, similar to the Kunc et al. study, which reported that ROS did not appear to impact cytotoxicity [44], ROS did not appear to impact the acute toxicity results in our studies. Thus, as mentioned above, it appears that a factor that could be influential in determining acute toxicity is agglomeration/aggregation, which may have been responsible for the similar MMAD values of the MPs and NPs in the inhalation studies.

Although our results suggest that NiO NPs, similarly to NiO MPs, do not exhibit acute or severe toxicity in rats via oral or inhalation exposure routes, there are still questions to be answered. For instance, what is the fate of these NPs (e.g., the uptake in local and secondary organs, macrophage phagocytosis, rate of excretion, etc.) once inhaled or ingested at the doses employed in our studies? What size are the NPs once inhaled or ingested? If they become agglomerated once inhaled or ingested, do they remain the same size or does further agglomeration or dissolution occur over time by physiological processes in the body? Future studies evaluating these questions could provide additional information on NiO NPs.

As a preliminary assessment, our studies were conducted according to the standard OECD guidelines for acute toxicity tests that were developed with non-nanoscale particles in mind. The OECD previously deemed “the approaches for the testing and assessment of traditional chemicals are in general appropriate for assessing the safety of nanomaterials but may have to be adapted to the specificities of nanomaterials” [45]. However, it is important to consider that nano-specific modifications (such as criteria/cutoff values for GHS classification, target MMAD for inhalation studies, adjustments to study protocols, etc.) for the acute toxicity testing of NMs could prompt the need for future follow-up studies.

## Figures and Tables

**Table 1:** Physico-chemical characteristics of the NiO test samples.

Test Substance	Purity (%)	BET Surface Area (m^2^/g)	Hydrodynamic Particle Size ^a^ (µm)	Shape	Reactive Oxygen Species ^b^ (nM)	Zeta Potential ^b^ (mV)	Dispersity Index ^b^
NiO NPs	99.9	74	0.210	Rectangular prism	23.8	−14.6	0.735
NiO MPs	96.4	96	15.6	NA	NA	NA	NA

^a^ Particle diameter (d0.5) was measured with Malvern Zetasizer (Malvern, Westborough, MA, USA; dynamic light scattering); NPs were measured in water; MPs had been previously measured in Isopar G, an organic solvent. ^b^ Analyses were evaluated in water. NA: not available.

**Table 2:** Details about the animals used in the acute studies.

Test Substance	Animal Species Strain	Source	Age (Weeks)	Initial Body Weight (Grams)	Acclimation Period (Days)
NiO NPs (oral and inhalation studies)	Rat Sprague-Dawley, derived albino	Charles River Laboratories	10–12	320–463, males 202–271, females	26–39
NiO MPs (low-dose inhalation study)	Rat Sprague-Dawley, derived albino	Ace Animals, Inc.	8–9	257–277, males 180–211, females	9
NiO MPs (high-dose inhalation study)	Rat Sprague-Dawley, derived albino	Harlan Laboratories, Inc.	8–9	222–246, males 158–172, females	7

**Table 3:** Acute oral exposure results.

Test Substance	Dose Level (mg/kg)	Mortality (# Dead/# Treated)	LD_50_ (mg/kg)
NiO NPs	175	0/1	>5000
	550	0/1	
	1750	0/1	
	5000	0/3	
Data below previously published in Henderson et al., 2012 [32]			
NiO MPs	5000	0/1	9990
	6300	0/1	
	7930	0/3	
	9990	2/4	
	11,000	2/2	

**Table 4:** Acute inhalation exposure results.

Test Substance	Nominal Conc. (mg/L)	Measured Conc. (mg/L)	MMAD (µm)	Mortality (# Dead/# Treated)		LC_50_ (mg/L)
				Male	Female	
NiO NPs	5.0	5.41–5.42	3.01–4.32	0/20	0/20	>5.42
NiO MPs	5.0	5.15	2.4–2.5	0/5	0/5	>8.30
	8.0	8.30	2.95–3.01	0/5	0/5	

## Data Availability

The data presented in this study are available upon request from the corresponding author.

## Supplementary Materials

The following supporting information can be downloaded at: https://www.mdpi.com/article/10.3390/nano13020261/s1: Table S1 contains the individual animal body weight observations following acute oral exposure to NiO NPs. Table S2 contains the individual animal body weight observations following acute oral exposure to NiO MPs (Henderson et al., 2012). Table S3 contains the individual in-life behavioral and necropsy observations following acute oral exposure to NiO NPs. Table S4 contains the individual in-life behavioral and necropsy observations following acute oral exposure to NiO MPs (Henderson et al., 2012). Table S5 contains the individual body weight observations following acute inhalation exposure to NiO NPs. Table S6 contains the individual body weight observations following acute inhalation exposure to NiO MPs. Table S7 contains the individual in-life behavioral and necropsy observations following acute inhalation exposure to NiO NPs. Table S8 contains the individual in-life behavioral and necropsy observations following acute inhalation exposure to NiO MPs.
