# Supplementary material for: An Assessment of the Oral and Inhalation Acute Toxicity of Nickel Oxide Nanoparticles in Rats
Source: Nanomaterials (Basel). 2023 Jan 7;13(2):261. doi: 10.3390/nano13020261 (PMC9860552; doi:10.3390/nano13020261)
Supplement: Supplementary file 1 [file nanomaterials-13-00261-s001.zip › nanomaterials-2139867-Supplemental Information.pdf]

ACUTE ORAL DATA - Individual Animal Body Weight

Table S1. Individual body weight observations following acute oral exposure to nickel oxide nanoparticles

Animal	Dose Level (mg/kg)	Body Weight (g)		
		Initial	Day 7	Day 14
Female 1	175	223.8	253.7	272.9
Female 2	550	229.2	248.7	280.7
Female 3	1750	217.4	236.7	271.4
Female 4	5000	214.7	246.5	282.6
Female 5	5000	205.5	236.7	254.8
Female 6	5000	210.5	241.6	251.2

Table S2. Individual body weight observations following acute oral exposure to nickel oxide micron particles (Henderson et al 2012)

Animals and Dosing Sequence[1]	Dose Level (mg/kg)	Body Weight (g)		
		Initial	Day 7	Day 14
Female 1	5,000	200	241	273
Female 2	6,300	208	230	274
Female 3	7,930	200	254	267
Female 8	7,930	198	234	248
Female 10	7,930	180	228	248
Female 4	9,990	175	178	232
Female 7	9,990	194	-	-
Female 9	9,990	185	-	-
Female 11	9,990	200	224	244
Female 5	11,000	179	-	-
Female 6	11,000	171	-	-

[1]Listed in order of increasing dose level instead of dosing sequence for easier comparison of results. Dosing sequence was based on the survival of the previous animal, according to the acute oral toxicity Up and Down Procedure.

ACUTE ORAL DATA - Individual Animal In-Life Behavioral and Necropsy Observations

Table S3. Individual in-life behavioral and necropsy observations following acute oral exposure to nickel oxide nanoparticles

	Individual Observations After Acute Oral Exposure to Nickel Oxide Nanoparticles																	
Animals	In-Life																	Necropsy
	Day 0 ½ hr	Day 0 3 hr	Day 0 3.5 hr	Day 1	Day 2	Day 3	Day 4	Day 5	Day 6	Day 7	Day 8	Day 9	Day 10	Day 11	Day 12	Day 13	Day 14	Gross Abnormalities
Female 1 175 mg/kg	AH	-	AH	AH	AH	AH	AH	AH	AH	AH	AH	AH	AH	AH	AH	AH	AH	None
Female 2 550 mg/kg	AH	-	AH	AH	AH	AH	AH	AH	AH	AH	AH	AH	AH	AH	AH	AH	AH	None
Female 3 1750 mg/kg	AH	AH	-	AH	AH	AH	AH	AH	AH	AH	AH	AH	AH	AH	AH	AH	AH	None
Female 4 5000 mg/kg	AH	AH	-	AH	AH	AH	AH	AH	AH	AH	AH	AH	AH	AH	AH	AH	AH	None
Female 5 5000 mg/kg	AH	-	AH	AH	AH	AH	AH	AH	AH	AH	AH	AH	AH	AH	AH	AH	AH	None
Female 6 5000 mg/kg	AH	-	AH	AH	AH	AH	AH	AH	AH	AH	AH	AH	AH	AH	AH	AH	AH	None

AH: Active and healthy

Table S4. Individual in-life behavioral and necropsy observations following acute oral exposure to nickel oxide micron particles (Henderson et al 2012)

	Individual Observations After Acute Oral Exposure to Nickel Oxide Micron Particles															
Animals[1]	In-Life															Necropsy
	Day 0	Day 1	Day 2	Day 3	Day 4	Day 5	Day 6	Day 7	Day 8	Day 9	Day 10	Day 11	Day 12	Day 13	Day 14	Gross Abnormalities
Female 1<br>5,000 mg/kg	AH	AH	AH	AH	AH	AH	AH	AH	AH	AH	AH	AH	AH	AH	AH	None
Female 2<br>6,300 mg/kg	AH	AH	AH	AH	AH	AH	AH	AH	AH	AH	AH	AH	AH	AH	AH	None
Female 3<br>7,930 mg/kg	AH	AH	AH	AH	AH	AH	AH	AH	AH	AH	AH	AH	AH	AH	AH	None
Female 8<br>7,930 mg/kg	AH	AH	AH	AH	AH	AH	AH	AH	AH	AH	AH	AH	AH	AH	AH	None
Female 10<br>7,930 mg/kg	AH	AH	AH	AH	AH	AH	AH	AH	AH	AH	AH	AH	AH	AH	AH	None
Female 4<br>9,990 mg/kg	AH	AH	AH	AH	AH	RF	RF	AH	AH	AH	AH	AH	AH	AH	AH	None
Female 7<br>9,990 mg/kg	AH	AH	RF	AH	-	-	-	-	-	-	-	-	-	-	-	Intestines discolored black/red
Female 9<br>9,990 mg/kg	AH	AH	RF	RF<br>FS	-	-	-	-	-	-	-	-	-	-	-	Intestines discolored black/red
Female 11<br>9,990 mg/kg	AH	AH	RF	RF	RF	AH	AH	AH	AH	AH	AH	AH	AH	AH	AH	None
Female 5<br>11,000 mg/kg	AH	AH	AH	AH	-	-	-	-	-	-	-	-	-	-	-	Intestines discolored black/red
Female 6<br>11,000 mg/kg	AH	AH	AH	RF<br>HO	-	-	-	-	-	-	-	-	-	-	-	Intestines discolored black/red

AH: Active and healthy; RF: reduced fecal volume; FS: facial staining; HO: hypoactivity; AG: ano-genital staining

[1] Listed in order of increasing dose level instead of dosing sequence for easier comparison of results. Dosing sequence was based on the survival of the previous animal, according to the acute oral toxicity Up and Down Procedure.

ACUTE INHALATION DATA - Individual Animal Body Weight

Table S5. Individual body weight observations following acute inhalation exposure to nickel oxide nanoparticles

Exposure Conc.	Animals	Individual Body Weight (g)				
		Initial	Day 1	Day 3	Day 7	Day 14
5.0 mg/L	Male 1	382.4	382.7	361.7	349.2	381.0
	Male 2	437.4	421.8	409.2	399.2	438.7
	Male 3	462.5	461.5	442.9	438.0	470.0
	Male 4	437.6	432.9	433.7	405.2	456.7
	Male 5	419.9	416.4	413.3	386.2	411.9
	Male 6	424.5	421.1	414.9	409.8	434.3
	Male 7	452.2	455.7	447.5	432.3	454.7
	Male 8	450.8	454.1	456.6	461.8	476.2
	Male 9	428.1	423.5	385.6	388.2	443.8
	Male 10	417.2	404.8	396.0	367.0	408.7
	Male 11	380.3	378.2	397.6	405.4	452.2
	Male 12	350.3	340.6	322.6	334.7	395.1
	Male 13	375.1	368.7	365.3	386.6	415.6
	Male 14	362.9	355.5	349.3	336.5	383.4
	Male 15	408.3	405.5	383.3	402.9	446.5
	Male 16	420.5	407.6	380.1	383.2	430.6
	Male 17	356.5	349.0	334.5	296.7	370.4
	Male 18	320.6	314.1	310.1	358.1	340.7
	Male 19	393.9	389.9	386.3	368.3	409.0
	Male 20	415.5	413.6	396.4	368.9	436.7
	Female 1	248.2	249.0	248.5	258.9	276.2
	Female 2	254.6	250.1	239.0	232.9	264.4
	Female 3	253.5	248.1	236.8	240.3	257.7
	Female 4	259.9	256.2	263.7	273.2	276.2
	Female 5	260.0	258.8	272.0	270.8	286.0
	Female 6	234.1	226.8	222.1	236.4	245.0
	Female 7	242.3	237.7	251.5	257.4	261.6
	Female 8	253.6	246.1	255.9	250.1	258.9
	Female 9	241.1	232.8	233.3	235.0	250.7

	Female 10	236.5	231.3	223.1	226.2	246.5
	Female 11	270.3	270.4	264.6	271.0	298.8
	Female 12	244.7	238.4	237.1	243.0	254.7
	Female 13	241.0	235.7	232.6	238.7	256.0
	Female 14	242.0	238.5	238.2	221.0	245.7
	Female 15	245.5	241.6	230.7	239.3	266.7
	Female 16	232.7	226.8	218.2	217.6	247.3
	Female 17	234.2	225.8	216.1	227.8	243.0
	Female 18	202.7	197.5	188.5	193.2	203.4
	Female 19	247.1	248.2	247.7	257.5	265.2
	Female 20	263.3	259.1	255.6	253.5	276.5

Table S6. Individual body weight observations following acute inhalation exposure to nickel oxide micron particles

Exposure Conc.	Animals	Individual Body Weight (g)				
		Initial	Day 1	Day 3	Day 7	Day 14
5.0 mg/L	Male 1	258	NR	NR	247	316
	Male 2	263	NR	NR	202	298
	Male 3	269	NR	NR	263	339
	Male 4	277	NR	NR	259	349
	Male 5	257	NR	NR	225	308
	Female 1	180	NR	NR	182	218
	Female 2	195	NR	NR	200	235
	Female 3	211	NR	NR	203	243
	Female 4	197	NR	NR	200	235
	Female 5	202	NR	NR	202	239
8 mg/L	Male 1	229	226	223	193	255
	Male 2	222	206	204	182	184
	Male 3	245	220	204	187	176
	Male 4	229	218	202	215	269
	Male 5	246	232	230	246	288
	Female 1	172	160	154	143	186
	Female 2	158	148	140	139	178
	Female 3	160	152	147	133	183
	Female 4	169	160	153	145	178
	Female 5	167	155	144	133	166

NR: not recorded

ACUTE INHALATION DATA - Individual Animal In-Life Behavioral and Necropsy Observations

Table S7. Individual in-life behavioral and necropsy observations following acute inhalation exposure to nickel oxide nanoparticles

Exposure Conc.	Animals	Individual Observations After Acute Inhalation Exposure to Nickel Oxide Nanoparticles																	
		In-Life																	Necropsy
		Day 0 CR[1]	Day 0 (1 hr)[2]	Day 0 (2 hr)[3]	Day 1	Day 2	Day 3	Day 4	Day 5	Day 6	Day 7	Day 8	Day 9	Day 10	Day 11	Day 12	Day 13	Day 14	Gross Abnormalities
5.0 mg/L	Male 1	IR	IR	IR	IR	IR	AH	IR	IR	AH	AH	AH	AH	AH	AH	AH	AH	AH	None
	Male 2	IR	IR	IR	IR	IR	IR	IR	IR	IR	IR	IR	AH	AH	AH	AH	AH	AH	None
	Male 3	IR	IR	IR	IR	IR	IR	IR	IR	IR	IR	AH	AH	AH	AH	AH	AH	AH	None
	Male 4	IR	IR	IR	IR	IR	IR	IR	IR	IR	IR	AH	AH	AH	AH	AH	AH	AH	None
	Male 5	IR	IR	IR	IR	IR	IR	IR	IR	IR	IR	AH	AH	AH	AH	AH	AH	AH	None
	Male 6	IR	IR	IR	IR	IR	IR	IR	IR	IR	IR	IR	AH	AH	AH	AH	AH	AH	None
	Male 7	IR	IR	IR	IR	IR	AH	AH	AH	AH	AH	AH	AH	AH	AH	AH	AH	AH	None
	Male 8	IR	IR	IR	IR	IR	IR	IR	IR	IR	IR	AH	AH	AH	AH	AH	AH	AH	None
	Male 9	IR	IR	IR	IR	AH	AH	AH	IR	IR	IR	AH	AH	AH	AH	AH	AH	AH	None
	Male 10	IR	IR	IR	IR	IR	IR	IR	IR	IR	IR	AH	AH	AH	AH	AH	AH	AH	None
	Male 11	IR	IR	IR	IR	IR	IR	IR	IR	IR	IR	IR	IR	IR	IR	IR	IR	IR	Lungs slightly discolored, spotted
	Male 12	IR	IR	IR	IR	IR	IR	IR	IR	IR	IR	IR	IR	IR	IR	IR	IR	IR	Lungs moderately discolored, spotted
	Male 13	IR	IR	IR	IR	IR	IR	IR	IR	IR	IR	IR	IR	IR	IR	IR	IR	IR	Lungs slightly discolored, spotted
	Male 14	IR	IR	IR	IR	IR	IR	IR	IR	IR	IR	IR	IR	IR	IR	IR	IR	IR	Lungs slightly discolored, red
	Male 15	IR	IR	IR	IR	IR	IR	IR	IR	IR	IR	IR	IR	IR	IR	IR	IR	IR	Lungs slightly discolored, red
	Male 16	IR	IR	IR	IR	IR	IR	IR	IR	IR	IR	IR	IR	IR	IR	IR	IR	IR	Lungs moderately discolored, spotted
	Male 17	IR	IR	IR	IR	IR	IR	IR	IR	IR	IR	IR	IR	IR	IR	IR	IR	IR	Lungs moderately discolored, red
	Male 18	IR	IR	IR	IR	IR	IR	IR	IR	IR	IR	IR	IR	IR	IR	IR	IR	IR	Lungs extremely discolored, spotted
	Male 19	IR	IR	IR	IR	IR	IR	IR	IR	IR	IR	IR	IR	IR	IR	IR	IR	IR	Lungs slightly discolored, red
	Male 20	IR	IR	IR	IR	IR	IR	IR	IR	IR	IR	IR	IR	IR	IR	IR	IR	IR	Lungs slightly discolored, red
	Female 1	IR	IR	IR	IR	IR	AH	AH	AH	AH	AH	AH	AH	AH	AH	AH	AH	AH	None
	Female 2	IR	IR	IR	IR	IR	AH	AH	AH	AH	AH	AH	AH	AH	AH	AH	AH	AH	None
	Female 3	IR	IR	IR	IR	IR	AH	AH	IR	AH	AH	AH	AH	AH	AH	AH	AH	AH	None
	Female 4	IR	IR	IR	AH	AH	AH	AH	IR	AH	AH	AH	AH	AH	AH	AH	AH	AH	None
	Female 5	IR	IR	IR	IR	IR	IR	IR	IR	AH	AH	AH	AH	AH	AH	AH	AH	AH	None
	Female 6	IR	IR	IR	IR	IR	IR	IR	IR	AH	AH	AH	AH	AH	AH	AH	AH	AH	None
	Female 7	IR	IR	IR	IR	AH	AH	AH	AH	AH	AH	AH	AH	AH	AH	AH	AH	AH	None
	Female 8	IR	IR	IR	IR	IR	IR	IR	IR	AH	AH	AH	AH	AH	AH	AH	AH	AH	None
	Female 9	IR	IR	IR	IR	AH	AH	AH	AH	AH	AH	AH	AH	AH	AH	AH	AH	AH	None

	Female 10	IR	IR	IR	IR	IR	AH	AH	IR	AH	AH	AH	AH	AH	AH	AH	AH	AH	None
	Female 11	IR	IR	IR	IR	IR	IR	IR	IR	IR	IR	IR	IR	IR	IR	IR	IR	IR	Lungs slightly discolored, red
	Female 12	IR	IR	IR	IR	IR	IR	IR	IR	IR	IR	IR	IR	IR	IR	IR	IR	IR	Lungs moderately discolored, red
	Female 13	IR	IR	IR	IR	IR	IR	IR	IR	IR	IR	IR	IR	IR	IR	IR	IR	IR	Lungs slightly discolored, spotted
	Female 14	IR	IR	IR	IR	IR	IR	IR	IR	IR	IR	IR	IR	IR	IR	IR	IR	IR	Lungs moderately discolored, red
	Female 15	IR	IR	IR	IR	IR	IR	IR	IR	IR	IR	IR	IR	IR	IR	IR	IR	IR	Lungs slightly discolored, spotted
	Female 16	IR	IR	IR	IR	IR	IR	IR	IR	IR	IR	IR	IR	IR	IR	IR	IR	IR	Lungs slightly discolored, red
	Female 17	IR	IR	IR	IR	IR	IR	IR	IR	IR	IR	IR	IR	IR	IR	IR	IR	IR	Lungs moderately discolored, red
	Female 18	IR	IR	IR	IR	IR	IR	IR	IR	IR	IR	IR	IR	IR	IR	IR	IR	IR	Lungs moderately discolored, red
	Female 19	IR	IR	IR	IR	IR	IR	IR	IR	IR	IR	IR	IR	IR	IR	IR	IR	IR	Lungs moderately discolored, spotted
	Female 20	IR	IR	IR	IR	IR	IR	IR	IR	IR	IR	IR	IR	IR	IR	IR	IR	IR	Lungs moderately discolored, spotted

IR: Irregular respiration; AH: Active and healthy

[1] Day 0 (CR) represents removal from the exposure tube following exposure

[2] Day 0 (1 hr) represents 1 hour after exposure

[3] Day 0 (2 hr) represents 2 hours after exposure

Table S8. Individual in-life behavioral and necropsy observations following acute inhalation exposure to nickel oxide micron particles

Exposure Conc.	Animals	Individual Observations After Acute Inhalation Exposure to Nickel Oxide Micron Particles																
		In-Life																Necropsy
		Day 0 CR[1]	Day 0 (1 hr)[2]	Day 1	Day 2	Day 3	Day 4	Day 5	Day 6	Day 7	Day 8	Day 9	Day 10	Day 11	Day 12	Day 13	Day 14	Gross Abnormalities
5.0 mg/L	Male 1	AH	AH	AH	AH	AH	AH	AH	AH	AH	AH	AH	AH	AH	AH	AH	AH	None
	Male 2	AH	AH	AH	AH	AH	AH	AH	AH	AH	AH	AH	AH	AH	AH	AH	AH	None
	Male 3	AH	AH	AH	AH	AH	AH	AH	AH	AH	HO	HO	HO	AH	AH	AH	AH	None
	Male 4	AH	AH	AH	AH	AH	AH	AH	AH	AH	HO	HO	HO	AH	AH	AH	AH	None
	Male 5	AH	AH	AH	AH	AH	AH	AH	AH	AH	HO	HO	HO	HO	AH	AH	AH	None
	Female 1	AH	AH	AH	AH	AH	AH	AH	AH	AH	AH	AH	AH	AH	AH	AH	AH	None
	Female 2	AH	AH	AH	AH	AH	AH	AH	AH	AH	AH	AH	AH	AH	AH	AH	AH	None
	Female 3	AH	AH	AH	AH	AH	AH	AH	AH	AH	AH	AH	AH	AH	AH	AH	AH	None
	Female 4	AH	AH	AH	AH	AH	AH	AH	AH	AH	AH	AH	AH	AH	AH	AH	AH	None
	Female 5	AH	AH	AH	AH	AH	AH	AH	AH	AH	AH	AH	AH	AH	AH	AH	AH	None
8.0 mg/L	Male 1	IR	IR	IR	AH	AH	AH	AH	PP	PP	PP	PP	PP	PP	PP	AH	AH	None
	Male 2	IR	IR	AH	AH	AH	AH	AH	AH	AH	IR<br>ND<br>HO	IR<br>ND<br>HO	IR<br>ND<br>HO	IR<br>ND<br>HO<br>RF	IR<br>HO<br>RF<br>PP	IR<br>RF	AH	None
	Male 3	IR	IR	AH	AH	AH	AH	AH	AH	PP	PP	IR<br>PP	IR<br>PP	IR<br>PP<br>RF	IR<br>PP<br>RF	PP	PP	Lungs, brown moderately dark spots
	Male 4	IR	IR	AH	AH	AH	AH	AH	AH	AH	AH	AH	AH	AH	AH	AH	AH	None
	Male 5	IR	IR	AH	AH	AH	AH	AH	AH	AH	AH	AH	AH	AH	AH	AH	AH	None
	Female 1	IR	IR	IR	AH	AH	AH	AH	HO	HO	AH	AH	AH	AH	AH	AH	AH	None
	Female 2	IR	IR	IR	IR	IR	AH	AH	AH	AH	AH	AH	AH	AH	AH	AH	AH	None
	Female 3	IR	IR	IR	IR	IR	AH	AH	HO	HO<br>AG	AH	AH	AH	AH	AH	AH	AH	None
	Female 4	IR	IR	AH	AH	AH	AH	AH	AH	AH	AH	AH	AH	AH	AH	AH	AH	None
	Female 5	IR	IR	AH	AH	AH	AH	AH	AH	HO	HO	AH	AH	AH	AH	AH	AH	None

IR: irregular respiration; AH: active and healthy; PP: prolapsed penis; ND: nasal discharge (red); HO: hypoactivity; RF: reduced fecal volume; AG: ano-genital staining

[1] Day 0 (CR) represents removal from the exposure tube following exposure

[2] Day 0 (1 hr) represents 1 hour after exposure
